# Survival Outcomes of Luminal Metastatic Breast Cancer Patients According to Changes in Molecular Subtype at Re-Biopsy: Insights from the GIM-13—AMBRA Study

**DOI:** 10.3390/cancers17101715

**Published:** 2025-05-20

**Authors:** Marina Elena Cazzaniga, Paolo Pronzato, Domenico Amoroso, Grazia Arpino, Francesco Atzori, Alessandra Beano, Laura Biganzoli, Giancarlo Bisagni, Livio Blasi, Cristina Capello, Rita Chiari, Alessia D’Alonzo, Michelino De Laurentiis, Angela Denaro, Alessandra Fabi, Daniele Farci, Francesco Ferraù, Elena Fiorio, Alessandra Gennari, Francesco Giotta, Filippo Giovanardi, Vanesa Gregorc, Lorenzo Livi, Emanuela Magnolfi, Anna Maria Mosconi, Raffaella Palumbo, Palma Pugliese, Carlo Putzu, Giuseppina Rosaria Rita Ricciardi, Ferdinando Riccardi, Laura Scortichini, Simon Spazzapan, Pierosandro Tagliaferri, Nicola Tinari, Giuseppe Tonini, Anna Maria Vandone, Giorgio Mustacchi

**Affiliations:** 1 Dipartimento di Medicina e Chirurgia, Università degli Studi Milano Bicocca, 20900 Monza, Italy; 2 Centro Ricerca Fase 1, Fondazione IRCCS San Gerardo, 20090 Monza, Italy; 3 Cancer Center IRCCS San Martino, 16100 Genova, Italy; pronza@tin.it; 4 UOC di Oncologia Medica, Ospedale Versilia, LU Azienda USL Toscana Nord Ovest, 55041 Lido di Camaiore, Italy; d.amoroso@usl12.toscana.it; 5 Azienda Ospedaliero Universitaria Federico II, 80131 Napoli, Italy; grazia.arpino@unina.it; 6 A.O.U. di Cagliari—Presidio Policlinico Duilio Casula, 09042 Cagliari, Italy; francescoatzori74@yahoo.it; 7 A.O.U. Città Della Salute e Della Scienza, 09042 Torino, Italy; alessandra.beano@gmail.com; 8 S.O.C. Oncologia Medica, Nuovo Ospedale di Prato Santo Stefano, Dipartimento Oncologico, Azienda USL Toscana Centro, 59100 Prato, Italy; laura.biganzoli@uslcentro.toscana.it; 9 Oncologia Medica Azienda USL-IRCCS di Reggio Emilia, 42122 Reggio Emilia, Italy; giancarlo.bisagni@ausl.re.it; 10 A.R.N.A.S. Ospedali Civico Di Cristina Benfratelli, 90127 Palermo, Italy; livio.blasi61@gmail.com; 11 Oncology Unit, Ospedale Martini della ASL Citta’ Di Torino, 10141 Torino, Italy; criscapello@libero.it; 12 UOC Oncologia—Azienda Sanitaria Territoriale Pesaro Urbino, 61121 Pesaro, Italy; rita.chiari@sanita.marche.it; 13 IRCCS Ospedale Policlinico San Martino, 16132 Genova, Italy; alessia.dalonzo@hsanmartino.it; 14 Istituto Nazionale Tumori IRCCS “Fondazione G. Pascale”, 80131 Napoli, Italy; m.delaurentiis@istitutotumori.na.it; 15 SC Oncologia Azienda Sanitaria Universitaria Giuliano Isontina (ASUGI) Trieste, 34148 Trieste, Italy; angela.denaro@asugi.sanita.fvg.it; 16 IRCCS Istituto Nazionale Tumori Regina Elena, 00128 Rome, Italy; alessandra.fabi@policlinicogemelli.it; 17 A.O. Brotzu—Ospedale Oncologico A. Businco, 09121 Cagliari, Italy; dr.daniele.farci@gmail.com; 18 UOC Oncologia Medica Ospedale San Vincenzo Taormina, 98039 Taormina, Italy; ferrau@oncologiataormina.it; 19 Oncologia Medica AOUI di Verona, 37126 Verona, Italy; elena.fiorio@aovr.veneto.it; 20 SCDU Oncologia, Azienda Ospedaliero-Universitaria “Maggiore della Carità” di Novara, 28100 Novara, Italy; alessandra.gennari@uniupo.it; 21 Struttura Complessa Oncologia Medica—IRCCS Istituto Tumori “Giovanni Paolo II”, 70124 Bari, Italy; francescogiotta@libero.it; 22 Medical Oncology, Comprehensive Cancer Centre, AUSL-IRCS di Reggio Emilia, 42122 Reggio Emilia, Italy; filippo.giovanardi@ausl.re.it; 23 Fondazione del Piemonte per l’Oncologia—Istituto di Ricovero e Cura a Carattere Scientifico (I.R.C.C.S.), 10060 Candiolo, Italy; vanesa.gregorc@ircc.it; 24 Department of Experimental and Clinical Biomedical Sciences “M. Serio”, Università di Firenze, 50134 Florence, Italy; lorenzo.livi@unifi.it; 25 Radiation Oncology Unit, Breast Unit—Florence University Hospital, 50134 Florence, Italy; 26 UOC Oncologia—PO SS Trinità Sora (Fr)—ASL FROSINONE, 03039 Frosinone, Italy; emanuela.magnolfi@libero.it; 27 Oncologia, Azienda Ospedaliera-Universitaria Perugia, 06156 Perugia, Italy; annamaria.mosconi@alice.it; 28 Oncologia Medica, Fondazione S. Maugeri IRCCS, 27100 Pavia, Italy; raffaella.palumbo@icsmaugeri.it; 29 Oncologia Medica, ASST Lariana, 2242 San Fermo della Battaglia, Italy; palma.pugliese@asst-lariana.it; 30 UOC Oncologia AOU Sassari, 07100 Sassari, Italy; cputzu@gmail.com; 31 Dipartimento di Onco-Ematologia, Azienda Ospedaliera Papardo, 98158 Messina, Italy; giusyricciardi81@hotmail.it; 32 Azienda Ospedaliera ‘A. Cardarelli’ (AORN), 80131 Napoli, Italy; nando.riccardi@gmail.com; 33 U.O. Oncologia, Ospedale di Macerata, 62100 Macerata, Italy; laura.scortichini@sanita.marche.it; 34 Medical Oncology and Cancer Prevention Unit, CRO Aviano National Cancer Institute IRCCS, 3381 Aviano, Italy; spazzapan@cro.it; 35 Dipartimento di Medicina Sperimentale e Clinica, Università Magna Graecia, 88100 Catanzaro, Italy; tagliaferri@unicz.it; 36 Policlinico «SS. Annunziata», 66100 Chieti, Italy; nicola.tinari@unich.it; 37 Medical Oncology, Fondazione Policlinico Universitario Campus Bio-Medico, 00128 Rome, Italy; g.tonini@policlinicocampus.it; 38 Oncologia Medica Ospedale Santa Croce e Carle, 12100 Cuneo, Italy; vandone.a@ospedale.cuneo.it; 39 University of Trieste, 4127 Trieste, Italy

**Keywords:** metastatic breast cancer, HER2 negative, biopsy, retesting

## Abstract

**Introduction**: The treatment of MBC patients is guided by receptor status, with re-biopsy at relapse recommended to reassess hormone receptor (HR) status. Various treatment options are available for HER2-veMBC, including CDK4/6 inhibitors, PARP inhibitors, and checkpoint inhibitors. The study highlights the importance of determining receptor subtype for guiding treatment choices. **Patients and Methods**: The GIM 13 AMBRA study is a longitudinal cohort study involving 42 centers in Italy. It includes data from 939 HER2- MBC patients enrolled between May 2015 and September 2020. The study analyzes the impact of HR expression changes on clinical outcomes using Kaplan–Meier survival curves and other statistical methods. **Results**: Among the 939 patients, 588 were rebiopsied at first relapse. The study found no significant differences in disease-free survival (DFS), progression-free survival (PFS), or overall survival (OS) between patients whose tumors changed molecular subtype and those who did not. However, post-progression survival from first-line treatment (PPS1) was different between the two groups. **Discussion**: The study confirms the phenomenon of receptor discordance between primary tumors and metastases. It emphasizes the need for re-biopsy in recurrent MBC to guide treatment strategies. The findings align with previous studies and highlight the importance of understanding receptor changes for improving patient outcomes. **Conclusions**: The GIM 13 AMBRA study provides valuable insights into the impact of molecular subtype changes on survival outcomes in Luminal MBC patients. It underscores the importance of re-biopsy and personalized treatment strategies in managing metastatic breast cancer.

## Simple Summary

Breast cancer is one of the most common oncological diseases among women in western countries and Italy as well. GIM 13-AMBRA is a patient journey study regarding how the prognosis of metastatic breast cancer patients can change according to the change in molecular subtype at relapse.

## 1. Introduction

Breast cancer (BC) remains the leading cause of cancer death among women worldwide [1]. In Italy, 55,000 new diagnosed cases per year and 13,000 deaths have been recorded [2]. Despite the great progress achieved in the treatment of the primary tumor, about 30% of breast patients are destined to develop distant metastases [3]. Among the most important factors associated with disease recurrence and overall survival, nodal involvement, hormone receptor status, Human Epidermal growth factor receptor-2 (HER2) expression and proliferation index play key roles [4]. The clinical course of metastatic breast cancer (MBC) is very heterogeneous in terms of growth rate and response to systemic therapies; however, medical treatments remain a palliative cure. Median survival is about 2 years for some subtypes. Treatment of metastatic breast cancer (MBC) patients is mostly based on the receptor status [5], independently of the setting (adjuvant vs. metastatic) and the line of therapy. In Human Epidermal growth factor Receptor 2-negative (HER2-) patients, a first biopsy at the time of relapse is strongly recommended to reassess hormone receptor status, especially in HR+, considering the wide choice of multiple therapeutic agents [6]. If the determination of hormone receptors (estrogen receptor—ER and progesterone receptor—PR) in the primary tumor is clinically essential to define breast cancer subtypes, clinical outcome and the choice of treatment, a redefinition of HR status as well of HER2 status in the metastatic setting can be useful to refine the choice and is now mandatory to allow patients access to some drugs [7]. Liquid biopsy, a recent technique which allows identification of fragments of tumor cells or DNA circulating in the blood [7,8] has replaced tissue biopsy for detection of gene mutations; however, the latter method remains fundamental to reassessing HR and HER2 status. 

Retrospective and prospective studies suggest that there is substantial discordance in receptor status between primary and recurrent breast cancer. Despite this evidence and current recommendations, the acquisition of tissue from metastatic deposits is not routine practice everywhere. Therefore, therapeutic decisions for treatment in the metastatic setting are based on the features of the primary tumor. Reasons for this attitude include the invasiveness of the procedure and the unreliable outcome of biopsy, in particular for biopsies of lesions at complex visceral sites. 

In recent years, various treatment options have become available for HER2- MBC. In the first-line setting, these options have included Cycline-Dependent Kinases 4/6 inhibitors (CDK4/6i) in combination with aromatase inhibitors or tamoxifen for HR+ patients, [9,10,11,12], PARP inhibitors for patients with BRCA1/2 mutations [13,14], and checkpoint inhibitors for PD-L1-positive triple-negative disease (TNBC) [15]. Following these developments, it has become increasingly important to determine the receptor subtype of the tumor in guiding systemic treatment choices, even in second- and further-line settings due to the potential changes in HR and HER2 expression between primary tumor and metastatic sites, or before and after a first- or second-line treatment. A recent meta-analysis, including 39 prospective and retrospective studies found discordance rates in terms of estrogen receptor (ER), progesterone receptor (PR), and HER2 status between primary breast tumor and loco-regional or distant recurrences of 19% for ER, 31% for PR, and 10% for HER2 [16]. In terms of clinical implications, a change in receptor subtype often leads to an adjustment in the treatment strategy, which can also translate into different outcomes, as different authors have reported [17]. The GIM 13—AMBRA study is a longitudinal, cohort study aiming to describe therapeutic choices in HER2- MBC in the Italian real-life setting. The main objectives of the GIM 13-AMBRA study have been previously described [18] and are described here briefly. They include the description of the strategies used as first, second or subsequent lines of treatment in patients receiving at least one chemotherapy line (CHT), and the evaluation of possible correlations between the choice of treatment (both in the adjuvant phase and for metastatic disease) and patient characteristics (age, menopausal status, comorbidities), as well as the evaluation of adherence to the literature recommendations for therapeutic sequences in the clinical practice. Here, we report data regarding the impact of molecular subtype changes (lack of hormone receptors at the first biopsy for metastatic disease) on clinical outcomes, on real-word outcomes progression-free survival (rwPFS), time to treatment change (TTC) and overall survival (OS).

## 2. Patients and Methods

### 2.1. Study Design

The GIM 13 AMBRA is a longitudinal cohort study, which has collected data of the first 50 consecutive HER2- MBC patients, who started a first-, second- or subsequent line CHT between January 2012 and December 2016 at 42 centers in Italy. As the type of treatment can vary according to different factors, i.e., the presence of clinical trials, or the availability of in-house services like pathology, the centers have been selected from the 192 national Oncological Centers listed in the “Libro Bianco 2012 of the Italian Association of Medical Oncology—AIOM), according to the hospital type (Cancer Center, University Hospital or General hospital) and geographical distribution. This selection ensured that the data collected were representative of the Italian situation. The inclusion criteria were as follows: age ≥ 18 years; patients with HER2-negative metastatic breast cancer (Stage IV) who have received or not received endocrine therapy for metastatic disease and were candidates for first-line chemotherapy treatment in the years 2014–2016 (prospective cohort), or who have received first, second, or subsequent lines of chemotherapy for metastatic disease in the years 2012–2013 (retrospective cohort); availability of all information required by the study, including histology, hormone receptor status, grading, stage of disease at diagnosis, type of surgery for the primary tumor, type of adjuvant therapy (chemo- or hormone therapy), type of drug received as adjuvant therapy, date and site of relapse, type of treatment received for the first/second/third line of treatment of metastatic disease (chemo- or hormone therapy), type of chemotherapy regimen used and details of the drugs, date and site of disease progression; and able to provide written informed consent. The exclusion criteria included the following: metastatic disease at diagnosis, HER2-positive status, and participation in clinical trials. All the centers were authorized by their ethical committees (ECs), after the approval of the Coordinating Center Ethical Committee (Comitato Etico Brianza, approved 27 November 2014, Authorization N.1831).

### 2.2. Objectives

The primary objective of the main study was to describe the strategies in terms of first, second and subsequent lines of treatment in patients receiving at least one chemotherapy line (CHT) and the relative outcome parameters of 939 HER2-ve MBC patients. Taxanes-based regimens, w/o targeted agents, were the preferred first choice in both Luminal (30.2%) and TNBC (33.3%) patients. Briefly, the median PFS1 was 12.5 months (95% CI: 16.79–19.64), without any significant difference according to subtypes, while PFS2 was significantly shorter in TNBC patients (5.5 months, 95% CI: 4.3–6.5 vs. Luminal A—9.4, 95% CI: 8.1–10.7, and Luminal B—7.7 95% CI: 6.8–8.2, F-Ratio 4.30, *p* = 0.014). [13]. In the present analysis, we report the data regarding the impact of HR expression change or not measured at the first biopsy performed at relapse on the main outcome parameters in HR+/HER2- tumors.

### 2.3. Statistical Analysis

The clinical outcomes were real-world progression-free survival at first- (rwPFS1) or second-line treatment (rwPFS2), defined as the time between first/second-line therapy start and time to progression, according to investigator, or censored to date of latest news; time to treatment change of first- (TTC1) or second-line (TTC2) therapy, defined as the time between the start date, declared by the investigator, of first- or second-line treatment and the date, not defined a priori due to the observational design of the study, of the subsequent therapy start. The variables were analyzed in the different groups of patients according to whether the hormone receptor status changed at relapse. Normal probability plots have been used to identify substantive deviations from original evolution. This includes identifying outliers, skewness, kurtosis, the need for transformations, and mixtures. Analyses were carried out using NCSS^®^ 12 statistical Software 2018 (Kaysville, UT, USA). Continuous variables were evaluated with descriptive statistics (including number of patients, mean, standard deviation, median, minimum, 25th and 75th percentiles, maximum). Categorical variables were evaluated with frequency and percentage. Tumor subtypes were defined according to the definition provided by Prat et al. [19].

## 3. Results

### Patients’ and Tumor Characteristics at First Relapse

**Figure 1:**
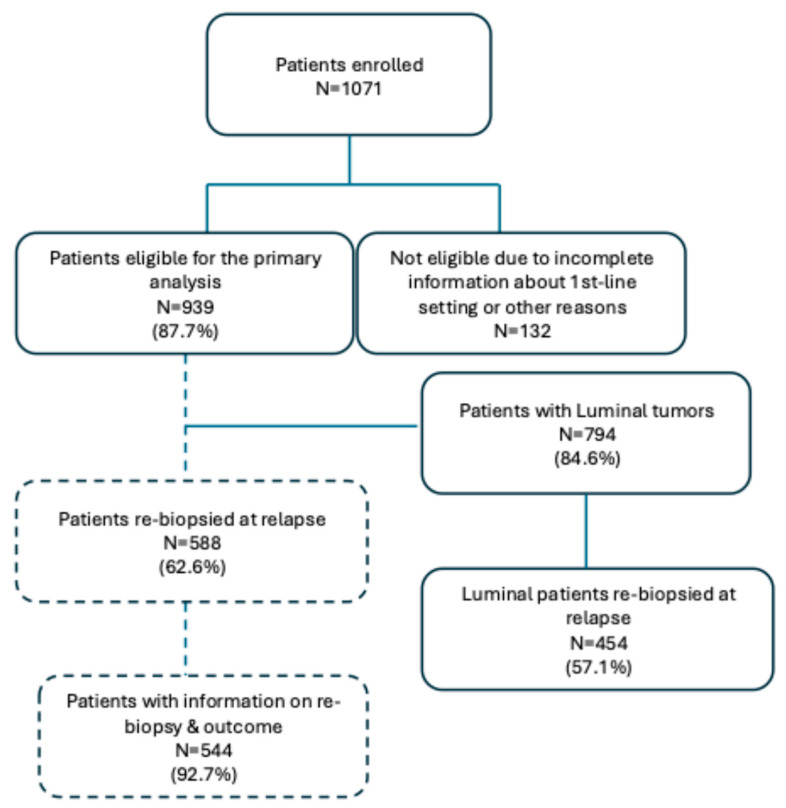
Consort diagram describes the enrolled population, and the final Luminal population considered for the present analysis.

Between May 2015 and September 2020, 1071 patients were enrolled in the main study, of whom 132 (12.3%) were not considered eligible due to (1) incomplete information about first-line treatment, and (2) other reasons, leaving a total of 939 (Luminal N = 794; TNBC, N = 145) evaluable patients for the main analysis. The demographic characteristics have been described in our previous paper. Briefly, median age at primary tumor diagnosis was 51.9 years (range 50.6–52.9), and most of the patients received adjuvant CHT (71.8%), mainly a combination of anthracycline + taxanes (305, 31.5%), or anthracycline + other drugs (266, 28.3%). Re-biopsy at relapse was performed in 588 out of 939 patients (62.6%) (Figure 1).

Percentages of tumors rebiopsed at relapse did not significantly differ according to the molecular subtype at primary diagnosis. More than 60% of the relapsed patients were retested for HR status at relapse. The details are reported in Table 1.

A total of 4 tumors (7.5%) among those rebiopsied changed their molecular category at relapse: among the Luminal tumors, 2 Luminal A became Luminal B and 31 became TNBC. Conversely, 11 TNBC tumors acquired a Luminal subtype at relapse. We did not collect data regarding change in HER2 status as all the patients enrolled were HER2-ve at the primary site. As previously declared and considering the occasional change into Luminal subtype occurring in TNBC tumors, as well as the unknown clinical relevance of this event, we report here outcome data only for Luminal tumors.

Five-hundred eighty-eight patients were retested for HR status determination at relapse; the 488 Luminal patients (134, 27.5%%) were treated with endocrine treatment in the adjuvant setting, either alone (134, 27.5%) or after chemotherapy (326, 66.8%), while the remaining were treated with chemotherapy alone (28, 5.7%%).

Most of the rebiopsied patients showed relapse at visceral sites (161, 32.9%) and bone (26.6%), as the non-rebiopsed one (visceral: 163, 46.4%; bone: 140, 39.9%. The majority of patients in both groups were treated with endocrine treatment at relapse, alone or sequenced after chemotherapy. Table 2 summarizes the type of treatments at relapse in retested and non-retested Luminal patients. Conversely, 11 TNBC tumors at primary acquired a Luminal profile at relapse: all patients but two were treated with chemotherapy, mainly a combination of Taxane + Bevacizumab.

No difference in terms of median disease-free survival (DFS) was observed in Luminal tumors according to the change in molecular subtype in comparison to tumors which did not change (Luminal: 74.6 months, 95% CI: 66.8–82.1 vs. Luminal becoming TNBC: 89.7 months, 95% CI: 44.7–103.5). Type of treatment was not significantly different between tumors with changed molecular subtype at relapse and tumors without change, as shown in Table 3.

**Figure 2:**
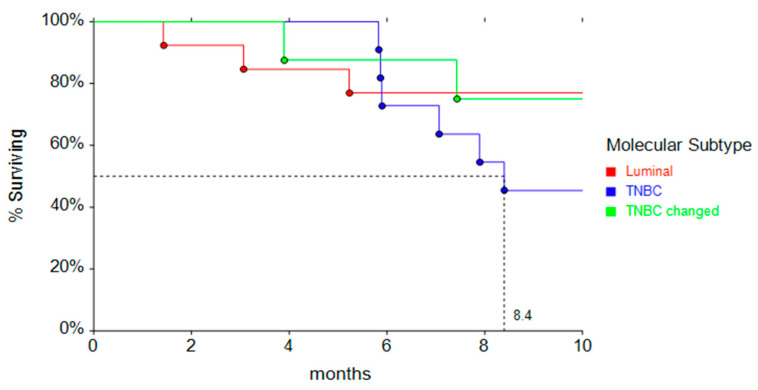
PFS Kaplan Meyer curves according to molecular subtype.

Conversely, 11 TNBC patients changed their molecular subtype, becoming Luminal at relapse. The median PFS1 in these patients is very similar to that observed in Luminal ones at primary (Figure 2).

Chemotherapy, alone or sequenced by ET, remained the most administered treatment, independently of the molecular subtype change or not.

**Figure 3:**
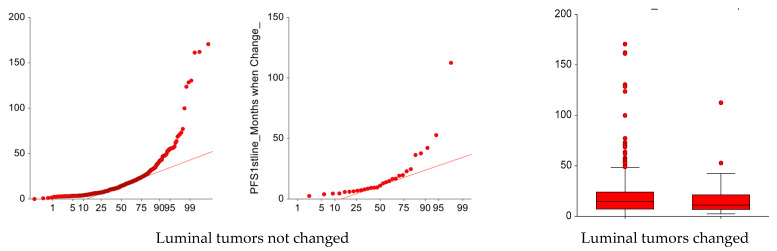
Normal probability plots of PFS1 according to the change in molecular subtype.

Subtype change did not significantly affect the main progression outcomes: no difference in median PFS1 was observed between the Luminal tumors which remain as such (14.4 months, 95% CI: 12.4–16) and those that changed into TNBC subtype (11.1 months, 95% CI: 7.2–16.8; *p* = 0.36) (Figure 3). 

**Figure 4:**
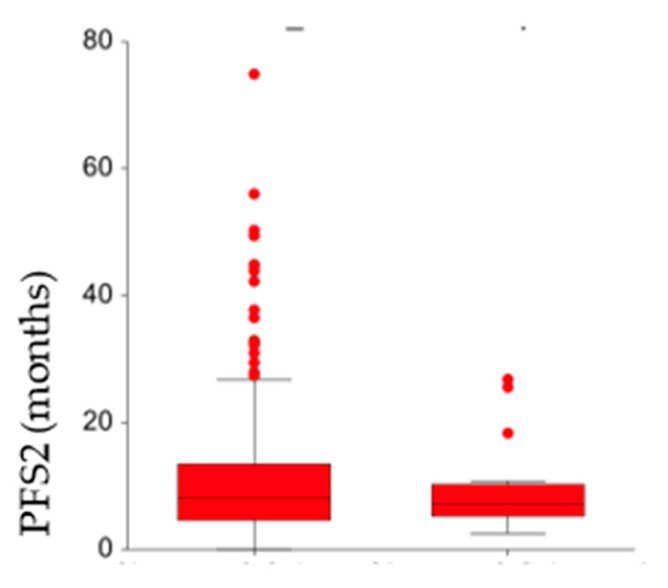
Normal probability plots of PFS2 (months) according to the change (or not) in molecular subtype.

For the two groups, the median PFS2 was 8.1 months (95% CI: 7.3–9.2) and 7.1 months (95% CI: 5.3–9.1), respectively, (*p* = 0.59) (Figure 4). 

**Figure 5:**
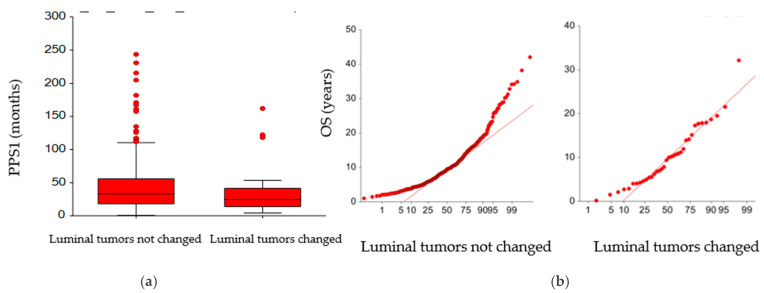
Normal probability plots of (**a**) PPS1 (months) and (**b**) OS (years) according to the change (or not) in molecular subtype.

Only median post-progression survival from first line treatment (PPS1) was different in the two groups (33.1 months, 95% CI: 30.1–36.6 vs. 24 months (95% CI: 15.8–30.9; *p* = 0.031) (Figure 5a), while no difference in median OS was observed between the two groups according to the change in molecular subtype (9.4 years, 95% CI: 8.7–9.8 vs. 9.7 years, 95% CI: 5.6–11.2) (Figure 5b).

## 4. Discussion

Discordance in ER, PgR and HER2 status between primary breast tumors and metastases is a well-known phenomenon, previously described by many authors and analyzed in different, even small, series [15,16]. However, the magnitude of this change in affecting patients’ management and survival outcomes remains important for defining therapeutic strategies, particularly in Luminal patients. The GIM 13-AMBRA study is a cohort, real-world study which collected data from more than 900 HER2- metastatic breast cancer patients in Italy from diagnosis until death, or data censoring, and so far, represents a priceless opportunity to collect insights useful for future trials. ESMO guidelines [2] strongly support the practice of having a biopsy for patients with recurrent MBC to confirm histology and to reassess ER, PgR and HER2 status. Among the 939 patients registered into the study, 588 (62.6%) were rebiopsied at first relapse, without any difference among Luminal A, Luminal B and TNBC patients. In a large retrospective study, Meegdes et al. [20] conducted a large real-world study which included all patients diagnosed with MBC between 2007 and 2018 in seven hospitals in the southeast Netherlands and registered in the SONABRE registry, with the aim of assessing the biopsy rate and the factors associated with taking a biopsy of a metastatic site at MBC diagnosis. Interestingly, they found that 60% of patients had a biopsy of a metastatic site at presentation, and the decision to have a biopsy was higher in academic hospitals (73%), in comparison to teaching hospitals (60%) and non-teaching hospitals (59%). The authors also found that a more recent period of MBC diagnosis was associated with a higher biopsy rate: 67% in 2016–2018 compared with 51% in 2007–2009 (OR = 2.14; 95% CI: 1.70–2.70). They also found that biopsy was performed more frequently in younger patients in comparison to older ones (56–75 years vs. >75 years: OR = 1.80; 95% CI: 1.48–2.19; ≤55 years vs. >75 years: OR = 2.20; 95% CI: 1.74–2.78)

The percentage of re-biopsies performed in our study is among the highest, if compared with also those reported in other retrospective studies, the largest one being the ESME database [16]. Grinda et al. reported that re-biopsy was performed in 17.6% of the 16,703 patients included in the ESME database. 

In our analysis, a total of 49 tumors (9.1%) among those rebiopsied changed their molecular category at relapse: 36 Luminal A or Luminal B tumors became TNBC (7.9%), and 13 TNBC became Luminal B (13%). Discordance rates between primary and metastases greatly varies according to the different series, strongly depending on the size of population analyzed. Meegdes et al. reported an overall receptor discordance rate of 18%; discordance rates were 13% for HR+/HER2- and 12% for TNBC. Mellouli et al. [21] collected data on 68 BC patients at a single-center in a retrospective study, reporting a discordance in ER status in 20 patients (29.4%, *p* = 0.041), with ER-negative conversion in 15 patients (22%) and ER-positive conversion in 5 patients (7.3%). They observed a difference in PR status in 27 of the cases (39.7%, *p* = 0.001): in 24 of the patients (35.2%), the PR status had changed from positive in PBC to negative in metastatic lesions, while in 3 of the patients (4.4%), the PR status had changed from negative to positive. HR status conversion was detected in 20 cases (29.4%, *p* = 0.04): 15 patients (22%) had changed from HR-positive status in primary breast cancer to HR-negative status in the metastatic tissues, and 5 patients (7.35%) from HR-negative to HR-positive. In the ESME cohort [22], one of the most important analyses ever conducted in a large population, regarding the discordance rate in HR expression between the primary site and the metastatic ones, Grind et al. reported a change rate for HR status of 14.2% [95% CI: 12.5–16.0], with expression loss in 72.5% and expression gain in 27.5%. For ER status, 15.1% [95% CI: 13.3–17.0] of cases showed a change with loss in 67.7% and gain in 32.3%. For PR status, a modification was observed in 31.1% [95% CI: 28.7–33.5] with loss in 75.3% and gain in 24.7%. To date, our results in terms of percentages of discordance between primary and metastases are in the middle of those reported by other authors [17], and this can be explained in part by the absence of a central review, as often happens in real-world studies, as well the unavailability of information regarding the site where the biopsy was taken. Other authors have thus showed that among the factors associated with HR discordance, there were metastases to bone only and primary tumor treatments with endocrine therapy [22].

The clinical meaning of discordance and, moreover, the impact in terms of treatment choice remains a matter of debate. In our analysis, we evaluated outcomes (rwPFS1, rwPFS2, PPS1 and OS) of 454 out of 490 rebiopsied Luminal patients, finding no differences in rwPFS1, rwPFS2 and OS. Differences among the various series remain wide, indicating there is probably the need for larger, prospective trials. Schwieger et al. [18] recently reported that the change from an ER or PgR+/HER2− at primary tumor to TNBC paired metastatic one was not associated with decreased survival (*p* > 0.05) in 258 analyzed patients. Similarly, Peng et al. [23] found no statistical difference in PFS according to the subtype of the recurrent or metastatic breast cancer (*p* > 0.05). In another retrospective study, Yang et al. [19] evaluated the frequency and the prognostic impact of changes in HR and HER2 between primary and recurrent/metastatic lesions in 133 patients: in their population, the ER-discordant cases and ER-loss cases experienced a worse overall survival (OS) (*p* = 0.001 and *p* = 0.016, respectively) and post-recurrence survival (PRS) (*p* = 0.001 and *p* = 0.018, respectively), compared with the respective concordant cases.

Testing for HER2 status is also important in that ~10% of HER2 status can change, including changing from negative to positive, due to clonal selection over time and therapeutic selection pressure. HER2 was not retested in our study, and we unfortunately do not know how often this occurred in this cohort, which is one of the limitations of our study. 

In the contemporary era, the treatment choice for HR+ BC which do not change hormone receptor expression from primary to metastases and for those which change into TNBC can significantly differ, both in terms of drugs, as well as in terms of additional molecular tests. For example, first-line treatment of TNBC patients is largely guided by the expression of programmed-death ligand 1 (PDL-1), while HR+ tumors may derive a benefit from more personalized approaches based on the detection of ESR1, AKT or PIK3CA mutations.

In recent years, different trials have evaluated the role of specific targeted agents, like elacestrant [7], imlunestrant [24], or inavolisib [25], as second-line treatment in advanced Luminal cancers, with the aim of delaying the use of chemotherapy. Overall, these trials demonstrated that, in the presence of specific gene mutations, they are highly efficacious in disease control. Nevertheless, most of them have been studied in patients who have been treated with CDK 4/6 inhibitors as first-line therapy and have received at least one prior chemotherapy line of treatment. Moreover, nothing is known about those patients excluded from enrollment in these trials and perhaps treated with chemotherapy.

Based on the evidence presented so far, when and how should we biopsy our metastatic patients? The availability of liquid biopsy, a technique which allows detection of breast cancer cell mutations in different organic fluids, mainly blood and urine, has revolutionized the approach to MBC patient treatment as it is easier to perform than tissue biopsies and is virtually non-invasive to the patient. However, it is our opinion that tissue biopsy should remain mandatory to assess ER and PgR status as a first approach, followed by a potential subsequent use of liquid biopsy to detect mutations specific to the molecular subtype. Several questions remain open: For example, considering the high heterogeneity of breast cancers, should we test for PDL-1 expression those Luminal tumors which lost HR expression, becoming TNBC at the time of relapse? How much is a biopsy obtained from a single metastatic site representative of the whole disease? Regarding these and other questions, answers are becoming more and more important, considering the high costs of the new drugs and, above all, the different safety profiles of the various drugs.

## Figures and Tables

**Table 1:** Proportion of tumors rebiopsied according to molecular subtype.

Luminal A	Luminal B	TNBC
234/386	254/408	100/145
60.6%	62.3%	68.9%

**Table 2:** Sites of relapse and type of treatment received in rebiopsed vs. non-rebiopsed Luminal patients.

	Rebiopsed Patients (N = 488)		Non-Rebiopsed Patients (N = 351)	
	**Sites of relapse**			
	N	%	N	%
Bone	102	20.9	122	34.7
Bone + soft tissue	28	5.7	18	5.1
Soft tissue	91	18.6	36	10.3
Visceral	114	23.4	82	23.4
Visceral + soft tissue	42	8.6	23	6.6
Visceral + bone	100	20.5	58	16.5
Visceral + bone + soft tissue	5	10.2	0	-
Other (non-visceral)	3	0.6	4	1.1
Not known	3	0.6	8	2.3
	**Type of treatment at relapse**			
Endocrine Therapy (ET)	178	36.5	156	44.4
Chemotherapy	159	32.6	112	31.9
Chemo → ET	151	30.9	83	23.6

**Table 3:** Type of treatment received for Luminal tumors which changed molecular subtype at relapse vs. tumors which maintained the original subtype.

	Luminal Tumors not Changed		Luminal Tumors Changed	
	N	%	N	%
	**Luminal A (N = 217)**		**Luminal A → Luminal B (N = 2)**	
ET	89	41.0	0	0.0
Chemotherapy	57	26.3	2	100.0
Chemo → ET	71	32.7	0	0
	**Luminal B (N = 238)**		**Luminal A&B → TNBC (N = 31)**	
ET	84	35.3	5	16.2
Chemotherapy	76	31.9	24	77.4
Chemo → ET	78	32.8	2	64.5

## Data Availability

Data supporting the reported results can be found at Oncotech.

## References

[1] Sung H., Ferlay J., Siegel R.L., Laversanne M., Soerjomataram I., Jemal A., Bray F. (2021). Global Cancer Statistics 2020: GLOBOCAN Estimates of Incidence and Mortality Worldwide for 36 Cancers in 185 Countries. CA A Cancer J. Clin..

[2] Cos’è il Cancro|AIRC. https://www.airc.it/cancro/informazioni-tumori/cose-il-cancro/numeri-del-cancro.

[3] Sørlie T., Perou C.M., Tibshirani R., Aas T., Geisler S., Johnsen H., Hastie T., Eisen M.B., Van De Rijn M., Jeffrey S.S. (2001). Gene expression patterns of breast carcinomas distinguish tumor subclasses with clinical implications. Proc. Natl. Acad. Sci. USA.

[4] Allison K.H. (2021). Prognostic and predictive parameters in breast pathology: A pathologist’s primer. Mod. Pathol..

[5] Curigliano G., Burstein H.J., Winer E.P., Gnant M., Dubsky P., Loibl S., Colleoni M., Regan M.M., Piccart-Gebhart M., Senn H.J. (2017). De-escalating and escalating treatments for early-stage breast cancer: The St. Gallen International Expert Consensus Conference on the Primary Therapy of Early Breast Cancer 2017. Ann. Oncol..

[6] Gennari A., André F., Barrios C.H., Cortes J., de Azambuja E., DeMichele A., Dent R., Fenlon D., Gligorov J., Hurvitz S.A. (2021). ESMO Clinical Practice Guideline for the diagnosis, staging and treatment of patients with metastatic breast cancer 5 behalf of the ESMO Guidelines Committee. Ann. Oncol..

[7] Bidard F.C., Kaklamani V.G., Neven P., Streich G., Montero A.J., Forget F., Mouret-Reynier M.A., Sohn J.H., Taylor D., Harnden K.K. (2022). Elacestrant (oral selective estrogen receptor degrader) Versus Standard Endocrine Therapy for Estrogen Receptor-Positive, Human Epidermal Growth Factor Receptor 2-Negative Advanced Breast Cancer: Results From the Randomized Phase III EMERALD Trial. J. Clin. Oncol..

[8] Mazzeo R., Sears J., Palmero L., Bolzonello S., Davis A.A., Gerratana L., Puglisi F. (2024). Liquid biopsy in triple-negative breast cancer: Unlocking the potential of precision oncology. ESMO Open.

[9] Slamon D.J., Diéras V., Rugo H.S., Harbeck N., Im S.A., Gelmon K.A., Lipatov O.N., Walshe J.M., Martin M., Chavez-MacGregor M. (2024). Overall Survival With Palbociclib Plus Letrozole in Advanced Breast Cancer. J. Clin. Oncol..

[10] Hortobagyi G.N., Stemmer S.M., Burris H.A., Yap Y.S., Sonke G.S., Hart L., Campone M., Petrakova K., Winer E.P., Janni W. (2022). Overall Survival with Ribociclib plus Letrozole in Advanced Breast Cancer. N. Engl. J. Med..

[11] Goetz M.P., Toi M., Huober J., Sohn J., Trédan O., Park I.H., Campone M., Chen S.C., Manso L.M., Paluch-Shimon S. (2024). Abemaciclib plus a nonsteroidal aromatase inhibitor as initial therapy for HR+, HER2- advanced breast cancer: Final overall survival results of MONARCH 3. Ann. Oncol..

[12] Lu Y.S., Im S.A., Colleoni M., Franke F., Bardia A., Cardoso F., Harbeck N., Hurvitz S., Chow L., Sohn J. (2022). Updated Overall Survival of Ribociclib plus Endocrine Therapy versus Endocrine Therapy Alone in Pre- and Perimenopausal Patients with HR+/HER2- Advanced Breast Cancer in MONALEESA-7: A Phase III Randomized Clinical Trial. Clin. Cancer Res..

[13] Robson M., Im S.A., Senkus E., Xu B., Domchek S.M., Masuda N., Delaloge S., Li W., Tung N., Armstrong A. (2017). Olaparib for Metastatic Breast Cancer in Patients with a Germline BRCA Mutation. N. Engl. J. Med..

[14] Robson M.E., Tung N., Conte P., Im S.A., Senkus E., Xu B., Masuda N., Delaloge S., Li W., Armstrong A. (2019). OlympiAD final overall survival and tolerability results: Olaparib versus chemotherapy treatment of physician’s choice in patients with a germline BRCA mutation and HER2-negative metastatic breast cancer. Ann. Oncol..

[15] Schmid P., Cortes J., Pusztai L., McArthur H., Kümmel S., Bergh J., Denkert C., Park Y.H., Hui R., Harbeck N. (2020). Pembrolizumab for Early Triple-Negative Breast Cancer. N. Engl. J. Med..

[16] Schrijver W.A.M.E., Suijkerbuijk K.P.M., Van Gils C.H., Van Der Wall E., Moelans C.B., Van Diest P.J. (2018). Receptor Conversion in Distant Breast Cancer Metastases: A Systematic Review and Meta-analysis. JNCI J. Natl. Cancer Inst..

[17] Kolberg-Liedtke C., Wuerstlein R., Gluz O., Heitz F., Freudenberger M., Bensmann E., du Bois A., Nitz U., Pelz E., Warm M. (2021). Phenotype Discordance between Primary Tumor and Metastasis Impacts Metastasis Site and Outcome: Results of WSG-DETECT-PriMet. Breast Care.

[18] Cazzaniga M.E., Pronzato P., Amoroso D., Bernardo A., Biganzoli L., Bisagni G., Blasi L., Bria E., Cognetti F., Crinò L. (2024). Clinical Outcomes of HER2-Negative Metastatic Breast Cancer Patients in Italy in the Last Decade: Results of the GIM 13-AMBRA Study. Cancers.

[19] Prat A., Adamo B., Cheang M.C.U., Anders C.K., Carey L.A., Perou C.M. (2013). Molecular characterization of basal-like and non-basal-like triple-negative breast cancer. Oncologist.

[20] Meegdes M., Ibragimova K.I., Lobbezoo D.J., Vriens I.J., Kooreman L.F., Erdkamp F.L., Dercksen M.W., Vriens B.E., Aaldering K.N., Pepels M.J. (2022). The initial hormone receptor/HER2 subtype is the main determinator of subtype discordance in advanced breast cancer: A study of the SONABRE registry. Breast Cancer Res. Treat..

[21] Mellouli M., Graja S., Kridis W.B., Ayed H.B., Makni S., Triki M., Charfi S., Khanfir A., Boudawara T.S., Kallel R. (2022). Discordance in receptor status between primary and metastatic breast cancer and overall survival: A single-center analysis. Ann. Diagn. Pathol..

[22] Grinda T., Joyon N., Lusque A., Lefèvre S., Arnould L., Penault-Llorca F., Macgrogan G., Treilleux I., Vincent-Salomon A., Haudebourg J. (2021). Phenotypic discordance between primary and metastatic breast cancer in the large-scale real-life multicenter French ESME cohort. NPJ Breast Cancer.

[23] Peng L., Zhang Z., Zhao D., Zhao J., Mao F., Sun Q. (2021). Discordance in ER, PR, HER2, and Ki-67 Expression Between Primary and Recurrent/Metastatic Lesions in Patients with Primary Early Stage Breast Cancer and the Clinical Significance: Retrospective Analysis of 75 Cases. Pathol. Oncol. Res..

[24] Jhaveri K.L., Neven P., Casalnuovo M.L., Kim S.B., Tokunaga E., Aftimos P., Saura C., O’shaughnessy J., Harbeck N., Carey L.A. (2024). Imlunestrant with or without Abemaciclib in Advanced Breast Cancer. N. Engl. J. Med..

[25] Turner N.C., Im S.A., Saura C., Juric D., Loibl S., Kalinsky K., Schmid P., Loi S., Sunpaweravong P., Musolino A. (2024). Inavolisib-Based Therapy in PIK3CA-Mutated Advanced Breast Cancer. N. Engl. J. Med..

